# An Efficient, Robust, and Inexpensive Grinding Device for Herbal Samples like *Cinchona* Bark

**DOI:** 10.3797/scipharm.1410-14

**Published:** 2015-02-19

**Authors:** Steen Honoré Hansen, Else Holmfred, Claus Cornett, Carla Maldonado, Nina Rønsted

**Affiliations:** 1 Analytical Biosciences, Dept. of Pharmacy, Fac. of Health and Med. Sci., Univ. of Copenhagen, Denmark; 2 Natural History Museum of Denmark, University of Copenhagen, Denmark

**Keywords:** Grinding, Bark and root, *Cinchona* bark, HPLC

## Abstract

An effective, robust, and inexpensive grinding device for the grinding of herb samples like bark and roots was developed by rebuilding a commercially available coffee grinder. The grinder was constructed to be able to provide various particle sizes, to be easy to clean, and to have a minimum of dead volume. The recovery of the sample when grinding as little as 50 mg of crude *Cinchona* bark was about 60%.

Grinding is performed in seconds with no rise in temperature, and the grinder is easily disassembled to be cleaned. The influence of the particle size of the obtained powders on the recovery of analytes in extracts of *Cinchona* bark was investigated using HPLC.

## Introduction

Sample preparation is an important first step in the chemical analysis of constituents in herbs. Typically, sample preparation involves pulverization and liquid extraction processes. The grinding of herbal samples like bark and roots into a fine powder is necessary in order to obtain more homogeneous samples, which again will result in more reliable analytical data. A more homogeneous sample obtained by pulverization also allows for the use of less sample material for analysis.

Furthermore, larger particles or pieces of plant material may be difficult to moisten and therefore, extract quantitatively. A finely ground sample is thus preferred for analytical chemical investigations.

The grinding of herbal samples like bark and roots can be performed in several ways by using a mortar and pestle, crushing after freezing in liquid nitrogen (~cryogenic grinding) [1, 2], in a ball mill, or other kinds of milling machines [3]. When using the first method, it is difficult to obtain a very fine and homogeneous powder and it is not possible to control the particle size of the final powder. The two latter techniques require relatively expensive equipment.

In the present paper, it is described how to modify a commercial coffee grinder into an effective and robust device that is able to handle and grind bark or root samples as small as 50 mg with a good recovery. The influence of the particle size on the extraction efficiency is also investigated. Although larger amounts of a sample can be ground in this device, the focus has been on the development of a device which could grind small samples with a reasonable recovery to be used also with herbarium specimens where possible sample sizes are limited. Additionally, the grinder is designed to be used for analytical problems including the need for grinding hundreds of bark or root samples requiring efficiency and speed in the grinding process.

The device is able to grind small bark samples of 0.05 g to 2.0 g within less than 10 sec and with a recovery > 60% at the 0.05 g level.

## Results and Discussion

### The Grinder

The Rancilio coffee grinder was modified in order to minimize the dead volume as much as possible. This was of utmost importance in order to be able to have a reasonably high recovery when grinding samples as small as 50 mg. The cleaning of the grinder between samples to avoid carryover is another important item. This is solved by adding a pin to the upper cutter, which can then be disassembled within 10 sec.

The recovery of the ground sample was investigated by grinding different amounts of the same *Cinchona* bark and by weighing the collected powder (Table 1).

**Tab. 1:**
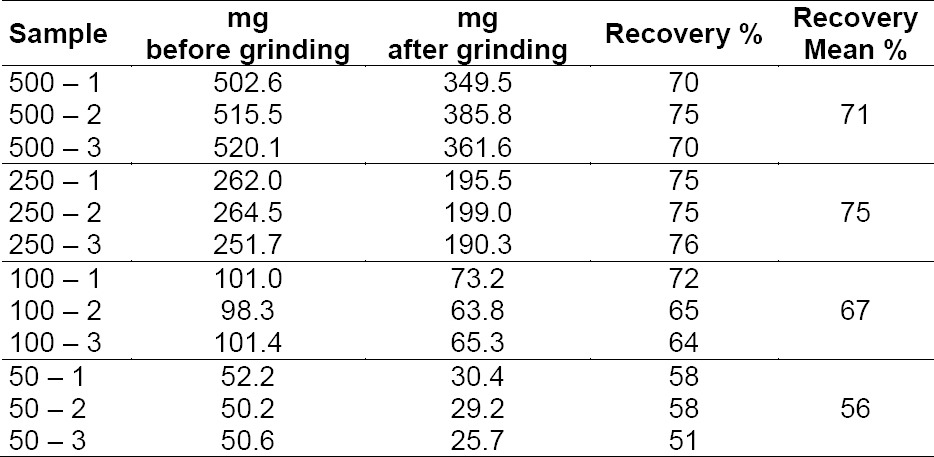
Recovery of *Cinchona* bark after grinding

It is seen that the recovery decreases with decreasing sample size. The main reason for the loss is that some of the sample is retained in the gratings of the two grinding wheels, which therefore have to be cleaned between samples in order to avoid carryover between samples. Even at the level of 50 mg of crude bark, about 30 mg of milled bark is recovered as a suitable powder for further analysis. For HPLC analysis, 100 mg of powder extracted to a volume of 10 mL is used, but this sample preparation may easily be scaled down by a factor of ten.

The important task is to obtain a fine powder without any larger particles. This will support an increase in the repeatability of the measurements, whereas powder with small and large particles together may result in less repeatable results when using small sampling sizes. A number of powders with various particle size distributions obtained by grinding the same *Cinchona* bark were analysed for the content of quinine by HPLC. For the extraction, a standard extraction solvent of 70% methanol containing 0.1% formic acid was used. No recovery studies were performed because only the effect of the particle size was to be evaluated.

The data given as peak areas in Tab. 2 show that samples with larger particles result in a lower extraction yield of the analyte (quinine). A typical chromatogram is shown in Fig. 3. The relative quantitative ratio between the four major alkaloids was constant in all samples measured.

**Tab. 2:**
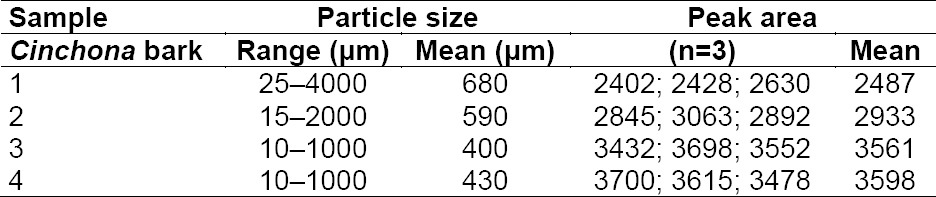
HPLC determination of quinine in *Cinchona* bark ground to four various particle sizes. Three separate determinations are performed on each sample

**Fig. 1:**
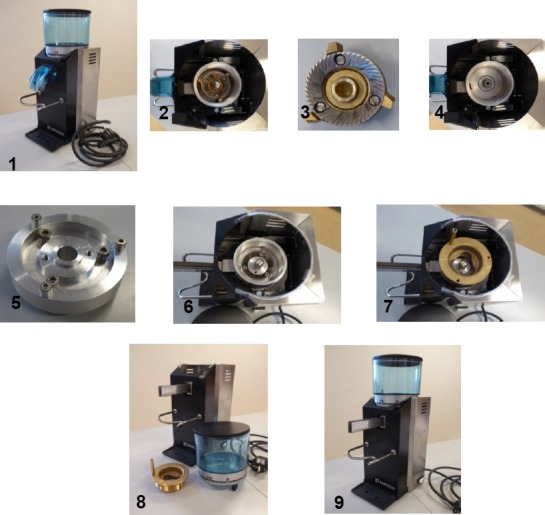
1) The purchased coffee grinder; 2) The grinder with the lower grinding wheel – observe the dead volume; 3) The lower grinding wheel; 4) The coffee grinder fully disassembled; 5) Zero dead volume insert; 6) Lower grinding wheel inserted in the zero dead volume unit; 7) Upper grinding wheel assembled; 8) The modified grinder dissembled; and 9) The modified grinding device

**Fig. 2:**
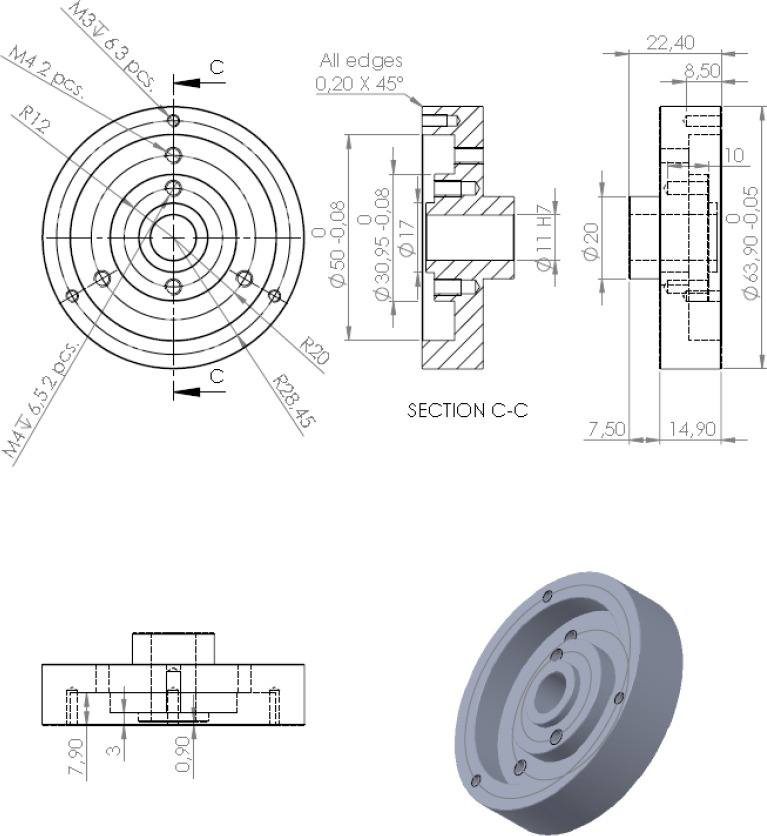
Technical drawing of the zero dead volume insert

**Fig. 3:**
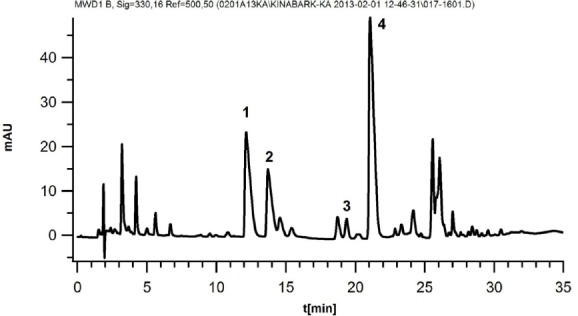
Chromatogram of an extract of Cinchona bark. 1) Cinchonine, 2) cinchonidine, 3) quinidine, and 4) quinine

When grinding samples, an increase in temperature in the sample may occur due to friction energy. Therefore, the temperature of the grinding wheels as well as the sample was measured before and after grinding. In all cases, no change in temperature within ± 1°C was detected (data not given). Sieving analysis was performed on sample 4 from Table 2 and on Rhei radix and Liquiritiae radix after grinding. The results are given in Table 3. A factor that may influence the fineness of the powder obtained is the degree of dryness of the sample.

**Tab. 3:**
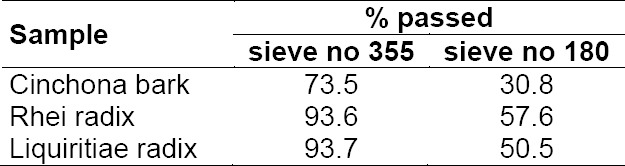
Sieving analysis of three samples after grinding

### HPLC

An HPLC system for the separation of the four major alkaloids (quinine, quinidine, cincho-nine, cinchonidine) in *Cinchona* bark as well as other constituents was developed. It was important that the system would also be compatible with mass spectrometric detection as this allows for better identification of alkaloids than UV. For HPLC, a modern solid core C18 column was chosen as it is known to deliver good peak symmetries. Acetonitrile was used as the organic modifier in most of the reversed-phase system for the separation of the *Cinchona* alkaloids published until now, giving a fair separation of all four major alkaloids [4–7]. However, a few experiments showed that the addition of methanol to the mobile phase improved the resolution between all four major alkaloids and thus, a mixture of acetonitrile + methanol (40:60 v/v) was used. The pH of the mobile phase was kept at 4.0 using 20 mM ammonium formate adjusted with formic acid. To be sure that all constituents were eluted from the column, gradient elution was performed. Detection was performed using UV at 330 nm where the major *Cinchona* alkaloids have an absorption maximum. Although this is not the strongest absorption, the choice of the longer wavelength improves the detection selectivity.

## Experimental

### Chemicals and Reagents

*Cinchona* cortex (Ph.Eur. quality) was obtained from Alfred Galke GmbH, Gittelde/Harz, Germany. Rhei radix was a gift from Naturmedicinsk Museum, University of Copenhagen and organic Liquiritiae radix was obtained from Naturdrogeriet A/S, Hørning, Denmark. Quinine sulfate was obtained from Merck (Darmstadt, Germany). All other chemicals were of HPLC or analytical grade quality and were obtained from Merck.

### The Grinding Device

A commercially available coffee grinder (Rancilio Rocky) was purchased from Rigtig Kaffe A/S, Skanderborg, Denmark (price in Denmark about 250 EUR). It was rebuilt in the following way in order to reduce the internal dead volume and to ease the assembling/disassembling of the grinding machine: an insertable alumina block (Figure 1.5 and Figure 2) was manufactured to fill out the dead volume around the lower cutter inside the grinder. The manufacture of the insert according to Figure 2 took about 2–3 hours by a precision mechanic for a cost of materials of about 30–40 EUR.

In the upper cutter wheel, a pin is inserted to ease the assembling/disassembling of the cutter in order to be able to clean the inside. For sample collection, a stainless steel chute is inserted into the outlet with zero dead volume.

A technical drawing of the zero dead volume insert is shown in Figure 2.

### HPLC

The HPLC system consisted of an Agilent 1200 system consisting of an on-line degasser, a binary pump G1312B, an autosampler G1367C, a column oven G1316B, and a DAD detector G1315C. The analytical separation column was a Kinetex XB-C18 (150 x 2.1 mm) with 2.6 μm particles. The gradient elution was performed using mobile phase A: 0.2 M ammonium formate buffer pH 4.0 and water (10:90 v/v) and mobile phase B: acetonitrile and methanol (40:60 v/v) at a flow rate of 0.2 ml/min. The gradient was 18% B from 0-10 min, then changed from 18% B to 36% B from 10-25 min returning to 18% B after 26 min with a total run time of 35 min. For UV detection, the wavelength at 330 nm was used.

### Sample Preparation for Grinding

Typically, the bark samples are cut into pieces the size of a coffee bean or smaller, and are transferred to the grinder. The fineness of the powder to be produced is determined by the tunable upper cutter. The grinding process takes typically less than 10 seconds.

### Sample Preparation for HPLC

One hundred mg of ground *Cinchona* bark was added to 2.0 mL of 70% methanol containing 0.1% formic acid. The mixture was ultrasonicated for 10 min and then diluted to 10.0 mL with mobile phase A. After centrifugation at 10,000 g, 5 μl was injected into the HPLC.

### Particle Size and Temperature Measurements

The particle size was measured on a Mastersizer 2000, Malvern Instruments, Worcestershire, UK. The temperature measurements were performed using a FLUKE, 62 Mini IR thermometer, Optris, RS Components A/S, Copenhagen, Denmark.

## Conclusion

An efficient and inexpensive grinding device for the disintegration of herbals has been constructed on the basis of a commercially available coffee grinder.

Very fine and homogenous powder can be obtained gently and with a uniform and repeatable particle size, which enables repeatable quantitative data even when using very small sample sizes. It is important to note that no increase in temperature was observed due to the very short grinding time in seconds. Furthermore, a new LC method for fingerprinting *Cinchona* bark is presented.
